# When to Reduce and Fix Displaced Lesser Trochanter in Treatment of Trochanteric Fracture: A Systematic Review

**DOI:** 10.3389/fsurg.2022.855851

**Published:** 2022-03-25

**Authors:** Ao-Lei Yang, Wei Mao, Jun-Guo Wu, Yi-Qun He, Hao-Fei Ni, Hai-Long Li, You-Hai Dong

**Affiliations:** ^1^Department of Orthopedics, Shanghai Fifth People's Hospital, Fudan University, Shanghai, China; ^2^Department of Orthopedic Surgery, Yangpu Hospital, Tongji University School of Medicine, Shanghai, China

**Keywords:** trochanteric fracture, lesser trochanter, reduce, fix, hip function, complication

## Abstract

**Purpose:**

To systematically evaluate the benefits of reducing and fixing displaced lesser trochanter (LT) of trochanteric fractures and when this procedure is worth the effect.

**Methods:**

From database establishment through March 2021, four online databases (PubMed, Cochrane, Embase, and Web of Science) were searched for relevant literature that investigated reduction and fixation for displaced LT of trochanteric fractures. The papers were then screened by two reviewers independently and in duplicate according to prior inclusion and exclusion criteria. Demographic data as well as data on fracture types, surgical protocols, and surgical outcomes were recorded, analyzed, and interpreted.

**Results:**

Total 10 clinical studies with 928 patients were included, in which 48 cases had intact LT and 880 cases involved the displaced LT, of which 196 (22.27%) cases underwent reduction and fixation for LT while the rest of 684 (77.73%) cases not. In these studies, complications were evaluated as a more applicable predictive parameter for operation than postoperative hip function.

**Conclusion:**

It was beneficial to reduce and fix the displaced LT when one of the conditions below occurred: displacement distance of LT ≥2 cm, quantity of comminuted LT fragments ≥2, and range of LT fragments in medial wall ≥75%; the fracture line of LT fragments reaching or exceeding the midline of the posterior wall.

## Introduction

As the population aging increasingly, the incidence of hip fractures in senile people is obviously elevated (1). Trochanteric fractures account for a significant proportion of hip fractures, ranging from 45 to 50%, in which 50–60% are classified unstable (2). According to the most used classification systems, Arbeitsgemeinschaft fur Osteosynthesefragen foundation and the Orthopedic Trauma Association (AO/OTA) classification and Evans-Jensen classification, this pattern of fracture may involve four fragments the distal femoral fragment, the femoral neck, the greater trochanter, and the lesser trochanter (LT) (3). It is accepted that orthopedists should try to anatomically reduce and fix the two main fragments and greater trochanter (4, 5). Lesser trochanter, an important structure of the femoral posteromedial wall, plays a pivotal role in stress distribution and reconstruction stability in trochanteric fracture (6). However, the necessity of reducing and fixing LT fragments remains controversial (2–4).

**Figure 1:**
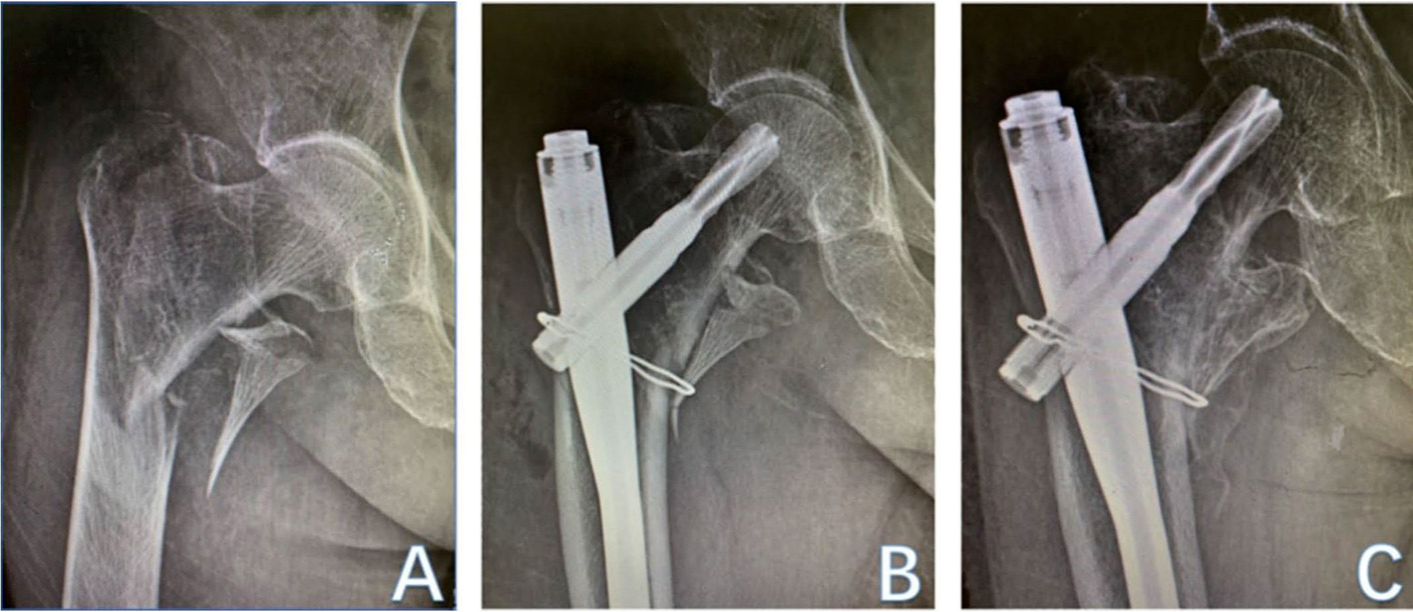
An 81-year-old woman suffered from trochanteric fracture with displaced LT **(A)**. Ten days later, she underwent operation of PFNA and cerclage wire **(B)**. The follow-up after 5 months implied LT union **(C)**.

At present, the most commonly used clinically operated protocols for trochanteric fracture include extramedullary fixation devices [e.g., dynamic hip screw (DHS)], intramedullary fixation devices[e.g., proximal femoral nail anti-rotation (PFNA)], and hip arthroplasty (e.g., hemiarthroplasty) (7, 8), but none of them are designed to fix the displaced LT. On the contrary, those orthopedic surgeons who advocate fixing the displaced LT have proposed kinds of fixation devices and skills since last century, including lag screw (9), cerclage wire (Figure 1) (2), double cables (10), candy-package (11), modified candy-package (12), etc.

**Figure 2:**
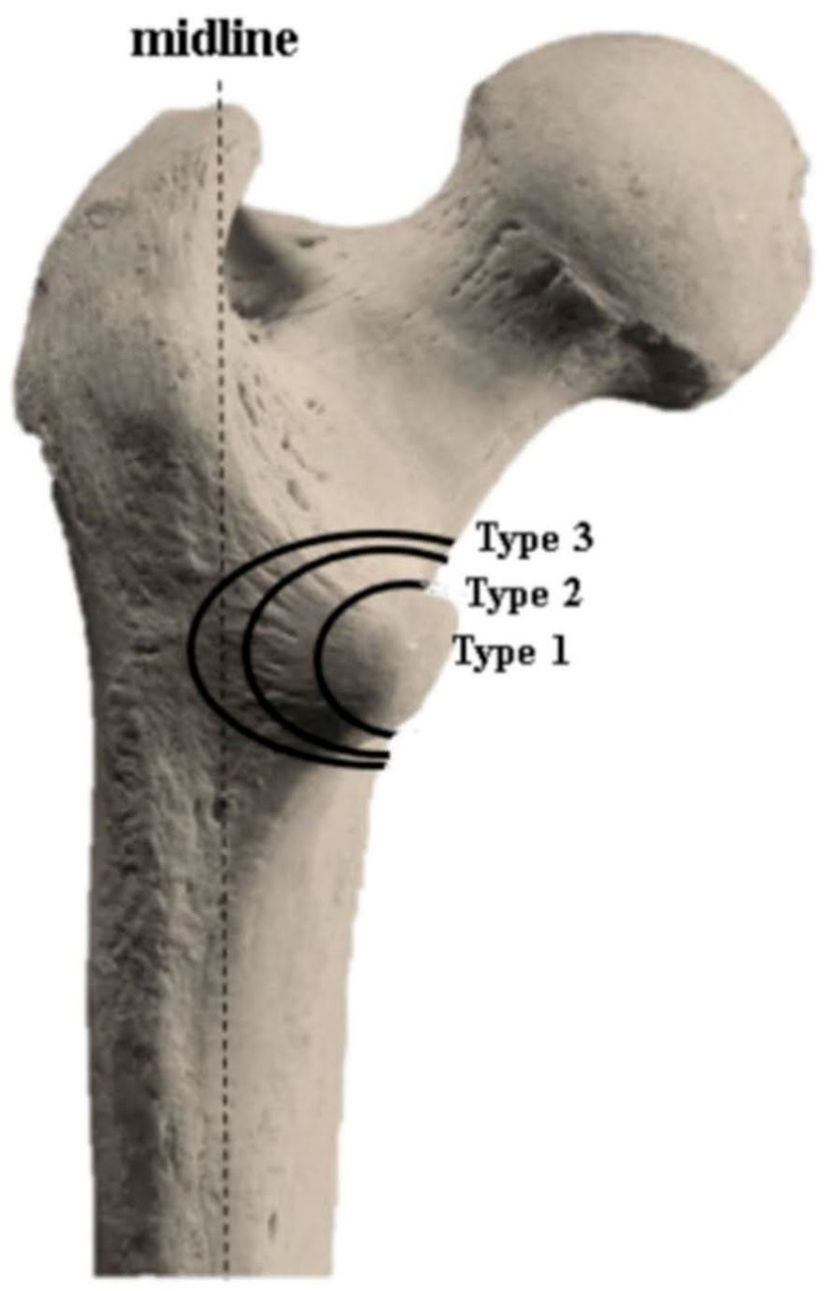
Classification for medial wall fracture.

Besides, most classifications, such as AO/OTA (1990) (13), revised AO/OTA(2018) (14), Evans (15), and Evans/Jensen (16), didn't investigated the stratification exclusive for LT fracturing degree. Recently, a novel classification (Figure 2) for medial wall fragments in trochanteric fractures has been proposed (17). It classified medial fragments into three types based on the degree of posterior cortex involvement: type 1: LT fragment with fracture line not exceeding base of the LT; type 2: a larger LT fragment and posterior cortex involved near the base of LT with fracture line not reaching the midline of the posterior wall; type 3: a much larger LT fragment and large posterior cortex involved with fracture line reaching or exceeding the midline of the posterior wall. To the best of our knowledge, this was the first study that classified the posteromedial cortex (mainly the LT) in trochanteric fracture and investigated its potential predictive role in complications.

With a high LT detachment ratio of over 50% in trochanteric fractures (3, 18), many reports tried to demonstrate whether it was worth the effort to reduce and fix the displaced LT. However, they couldn't reach a consensus. Some reports (2, 8, 19) implicated that LT fixation could regain higher primary stability, resulting in lower implants failure rates, but the incidental price may increase, such as longer operation time, increased intra-operative blood loss, and iatrogenic injury of the surrounding nerves/vessels. Therefore, they concluded that reduction and fixation for LT should be individually weighed upon the patients' mobility demands and health status. In the meanwhile, several studies (3, 20, 21) demonstrated that reducing and fixing LT or not would get similar outcomes. On the contrary, some studies (4, 17, 22) verified that severely displaced LT might increase the postoperative complications, from which they recommended that this kind of LT should be reduced and fixed. Moreover, it is noticed that orthopaedists paid more attention to the postoperative injury of surrounding vessels (23–27) especially potentially life and limb-threatening pseudoaneurysm (28–37), caused by displaced LT. Therefore, the question “does the reduction and fixation for LT fragment in trochanteric fracture treatment worth the effect?” is hardly to be answered.

## Methods and Materials

### Search Strategy

The current systematic review was performed using the Preferred Reporting Items for Systematic Reviews and Meta-Analyses (PRISMA) as the guidelines. From database establishment through November 2021, four online databases (PubMed, Cochrane, Embase, and Web of Science) had been searched that investigated reduction and fixation of LT in trochanteric fractures, with no language restriction (non-English literature was translated if necessary). The broad search included the following terms “lesser trochanter,” “trochanteric fracture,” “reduce,” “fix,” and “wire.” Moreover, the reference lists of the identified studies were hand-searched and gray literature sources, such as Open-Gray, were searched as additional search methods.

### Eligibility of Studies

Inclusion criteria were as follows: (1) investigating human trochanteric femur fractures; (2) existing LT fragment; (3) reporting postoperative outcomes associated with fixed or unfixed LT fragments; and (4) randomized controlled trials (RCT), cohort studies, case-control studies, and case series.

Exclusion criteria were as follows: (1) femoral neck fracture involved; (2) pathologic fracture of LT (tumor metastasis); (3) LT avulsion fractures in adolescents with high-energy violence; and (4) no full-text available.

### Screening Strategy

The initial outcome involved a total of 471 studies after searching four databases and other sources. The abstract and full text of this literature were screened by two reviewers (Yang and Mao) independently and in duplicate according to prior inclusion and exclusion criteria. Any screening discrepancies would be reassessed by the third senior reviewer (Dong) till they reached an agreement.

### Data Extraction

Data of all included studies were collected and recorded in an Excel spreadsheet (Version 2019; Microsoft Corp) by two reviewers (Yang and Mao), which included the information of the authors, published date, study design, sample size, mean age, mean follow-up, surgical protocols, and outcomes.

### Quality Evaluation for Included Studies

The methodological quality of all included studies was evaluated using Methodological Index for the Non-Randomized Studies (MINORS) instrument, the Newcastle-Ottawa Scale (NOS) instrument, and the Cochrane Risk of Bias (ROB) tool. MINORS are used to evaluate the non-comparative, comparative, and non-randomized studies with maximum scores of 16, 24, and 24, respectively (38). NOS is specially designed to assess the quality of case-control study and cohort study both with the maximum scores of 9 (39). Besides, the ROB tool is appropriate for the quality assessment of RCTs with the ROB figure (40).

### Statistics Analysis

The inter-reviewer agreements on abstract screening, full-text screening, and grading scores of the studies quality were evaluated using the kappa statistic. In the meanwhile, statistical analysis was performed using STATA (Stata Corp. LLC; College Station; Stata/MP 16.0 for Windows). It should be noted that owing to the inadequate reports, methodological difference, and various outcome indicators of included research, quantitative synthesis/meta-analysis was limited to be performed. Therefore, we performed the descriptive analysis for all included studies, and numeric data and categorical data were expressed as Mean ± SD and numbers with percentages, respectively. Statistics significance was calculated by Student's *t*-test, one-way ANOVA, and the Fisher exact test with a significance level of 0.05.

## Results

### Characteristics of Studies

**Figure 3:**
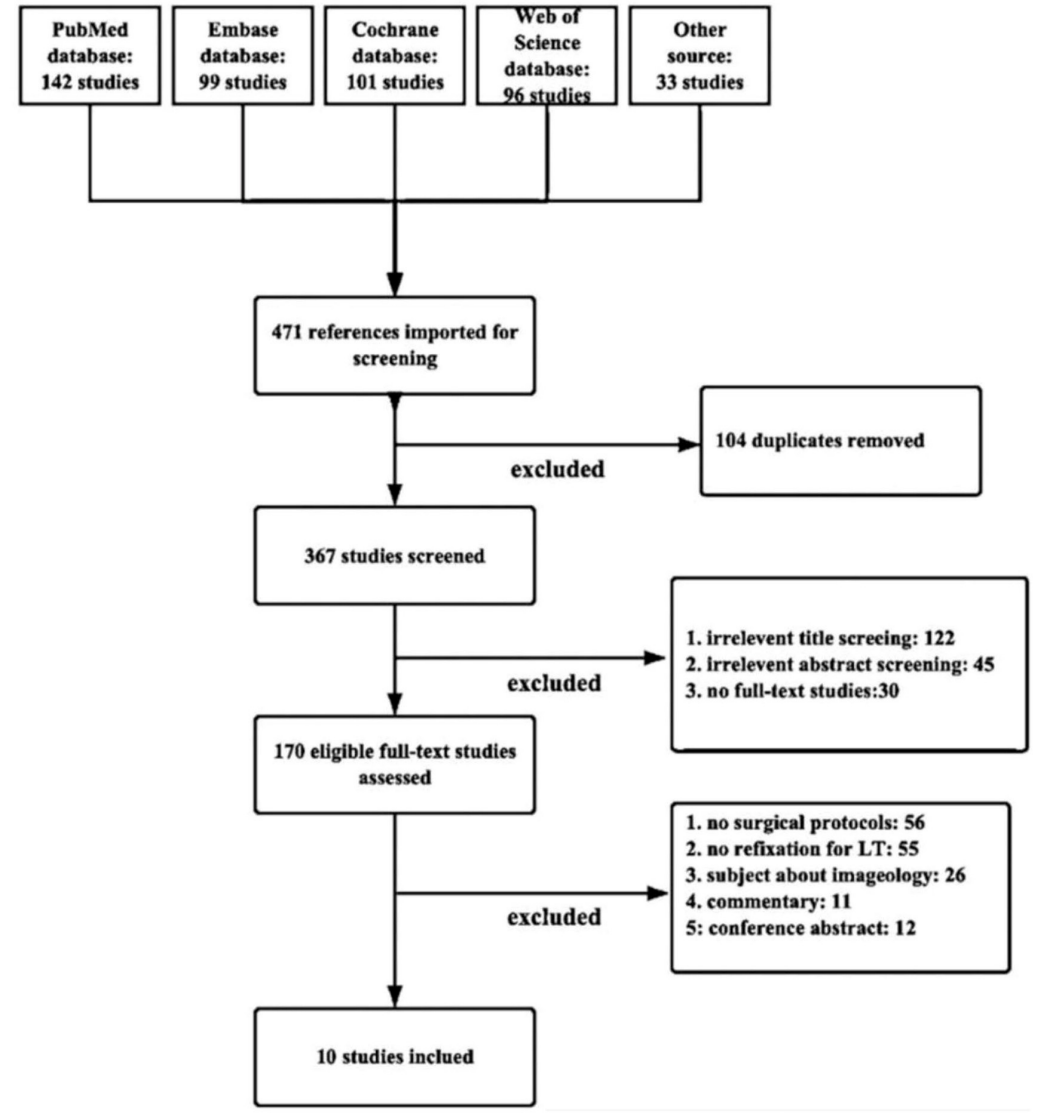
PRISMA (Preferred Reporting Items for Systematic Meta-Analyses) flow diagram demonstrating the systematic review of the literature for studies investigating fixation necessity of displaced lesser trochanter in trochanteric fractures.

The primary retrieve for online databases resulted in 471 studies, of which 10 were full-text studies that met the inclusion and exclusion criteria (Figure 3). The reviewers reached substantial agreement and the interreviewer reliability at the abstract (κ = 0.893 [95% *CI*, 0.889–0.897]), full-text (κ = 1.000), and the quality evaluation of studies (κ = 0.841 [95% *CI*, 0.801–0.881]) was available.

**Figure 4:**
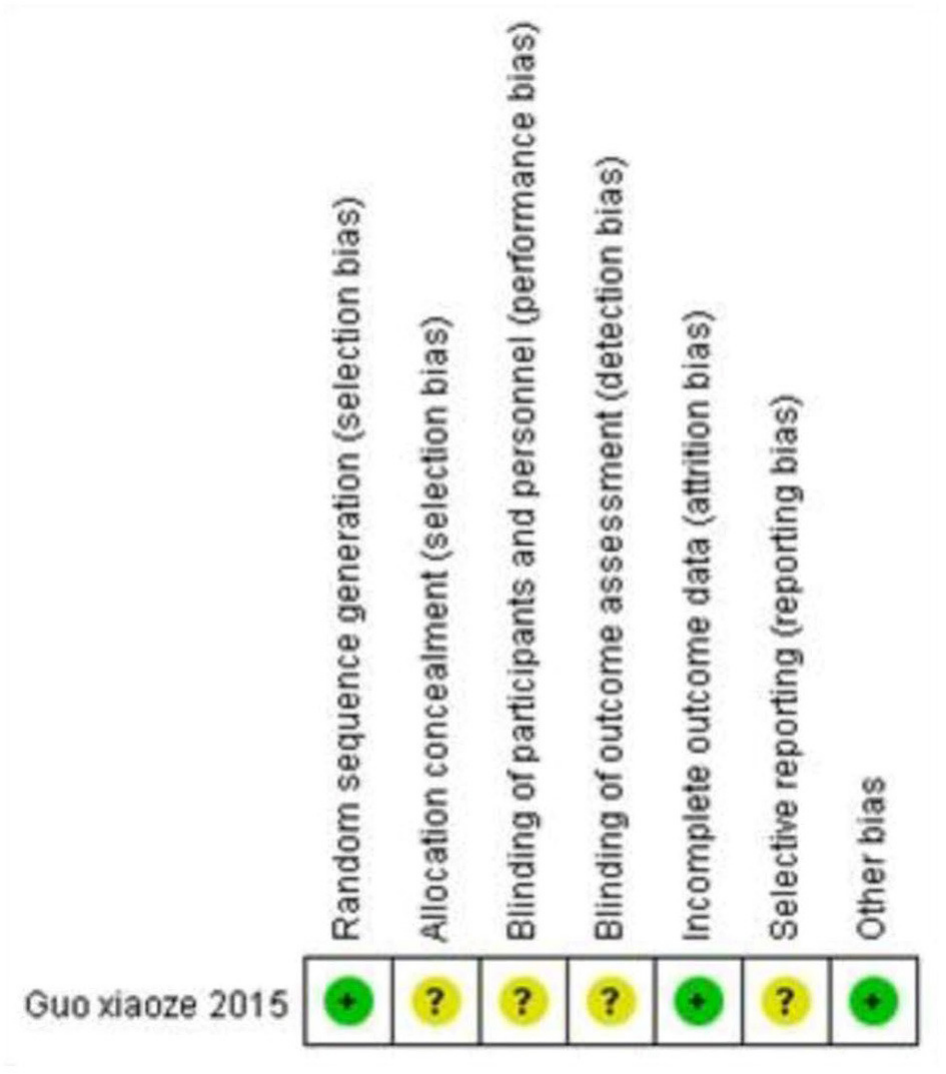
Risk of bias assessment for the lone included randomized controlled trial.

The included 10 studies (Table 1) consisted of two retrospective case series (Level 4), one prospective case series (Level 4), six retrospective comparative studies (Level 2), and one RCT (Level 1). The three cases series were evaluated by MINORS checklist with mean scores of 10.67 (range, 10–12), implicating a fair quality of evidence. The six retrospective comparative studies were assessed by NOS with mean stars of 7.83 (range, 6–9), demonstrating a high quality of evidence. In the meanwhile, using the ROB tool, evidence quality of the lone RCT was verified as a moderate risk of bias (Figure 4).

**Table 1:** Characteristics of included clinical studies.

**References**	**Study design (level of evidence)**	**No. of patients**	**Classification**	**LT fragment involved**	**Case treatment**	**Mean age, y**.	**Mean follow-up, mo**.	**Quality assessment**
Kim et al. (12)	Cases series (IV)	22	31-A2: 15 31-A3: 7	All with DDLT > 5 mm	IM nails with wiring	75.8	15.1	MINORS: 10
Sun et al. (22)	Retrospective comparative (II)	A: 42 B: 33 C: 36	A: 31-A2: 39 31-A3: 3 B: 31-A2: 30 31-A3: 3 C: 31-A2: 34 31-A3: 2	All A: DDLT <1 cm B: DDLT ≥1 cm without fixation C: DDLT ≥1 cm with fixation	A: IM nails B: IM nails C: IM nails with wiring	A: 77.7 B: 77 C: 78.3	A: 17.2 B: 17.6 C: 17.9	NOS: 9
Liu et al. (5)	Retrospective comparative (II)	A: 48 B: 37	A: 31-A2: 46 31-A3: 2 B: 31-A1: 36 31-A3: 1	A: All B: None	IM nails	A: 77.3 B: 78.1	Minimum: 1 year	NOS: 6
Ye et al. (9)	Case series (IV)	32	EVANS II: 12 EVANS III:13 EVANS IV: 7	All	19 DHS with lag screw 13 DHS with wiring	64	13	MINORS: 10
Guo et al. (41)	Randomized controlled trail (I)	A: 32 B: 34	A: EVANS III: 32 B: EVANS III: 34	All	A: DHS with LT fixator B: DHS	A: 59 B: 62	A: 15 B: 16	ROB (Figure 4)
Puram et al. (20)	Retrospective comparative (II)	102	31-A2	all	A: 28 DHS B: 74 DHS with wiring	72	20	NOS: 9
Aprato et al. (19)	Retrospective comparative (II)	A: 12 B: 11	A: 31-A2: 8 31-A3: 4 B: 31-A1: 7 31-A3: 4	A: All B: None	A: IM nails B: IM nails	A: 51.7 B: 58.7	A: 43.1 B: 40.8	NOS: 6
Schenkel et al. (3)	Cases series (IV)	20	31-A2 and 31-A3	All DDLT: 25 (20–47) mm	19 IM nails 1 Condylar plate	74	15.2	MINORS: 12
Li et al. (17)	Retrospective comparative (II)	324	31-A2.1: 259 31-A2.2: 33 31-A2.3: 32	A: type 1^a^ B: type 2^a^ C: type 3^a^	A: 186 IM nails B: 76 IM nails C: 62 IM nails	73.2	27.3	NOS: 8
Ren et al. (42)	Retrospective comparative (II)	143	31-A2	A: LTFQ^b^ = 1 B: LTFQ = 2 C: LTFQ > 2	A: 48 IM nails B: 52 IM nails C: 43 IM nails	73.5	16.1	NOS: 8
				D: LTFR^b^ <75% E: LTFR ≥ 75%	D: 62 IM nails E: 81 IM nails			

*Data are shown as mean, mean (range), mean ± SD, or mean ± SD (range)*.

a *type 1: LT fragment with fracture line not exceeding base of the lesser trochanter; type 2: a larger fragment of LT and posterior cortex involved near the base of LT with fracture line not reaching the midline of the posterior wall; type 3: a much larger fragment of LT and large posterior cortex involved with fracture line reaching or exceeding the midline of the posterior wall*.

b *LTFQ: quantity of comminuted lesser trochanter fragments. LTFR: range of lesser trochanter fragment in the medial wall*.

*NR, not reported*.

In each of the comparative studies, statistical differences of demographic data (such as gender ratio, age distribution ratio, and follow-up ratio) between/among groups were calculated, which ensured baseline groups equivalence.

### Demographic and Clinical Characteristics

Based on the limited studies involving the topic, only 10 reports were identified in the current study (Table 1). These studies described a total of 928 patients, from which 48 (5.17%) cases had intact LT while 880 (94.83%) cases involved the displaced LT, of which 196 (22.27%) cases underwent fixation of LT while the rest of 684 (77.73%) cases not. Moreover, these 928 patients with a mean age of 70.36 years (range, 23–94 years) comprised 389 men and 539 women. Besides, the mean follow-up was 20.15 months (range, 6.5–57 months) with eight studies reporting a minimum follow-up of 1 year except for the shorter follow-up of 6.5 months (3) and 9 months (9) in two reports. Besides, nine studies had an attrition ≤ 25.00% while one study (5) had a large attrition of 33.07%. Due to variations of reported outcomes among included studies, including eight studies reporting postoperative hip function, two (5, 41) reporting surgical costs, two (3, 19) reporting hip flexion strength, three (12, 41, 42) reporting change of neck-shaft angle (NSA), and four (5, 9, 12, 22) reporting complication incidence.

### Characteristics of Lesser Trochanter

**Figure 5:**
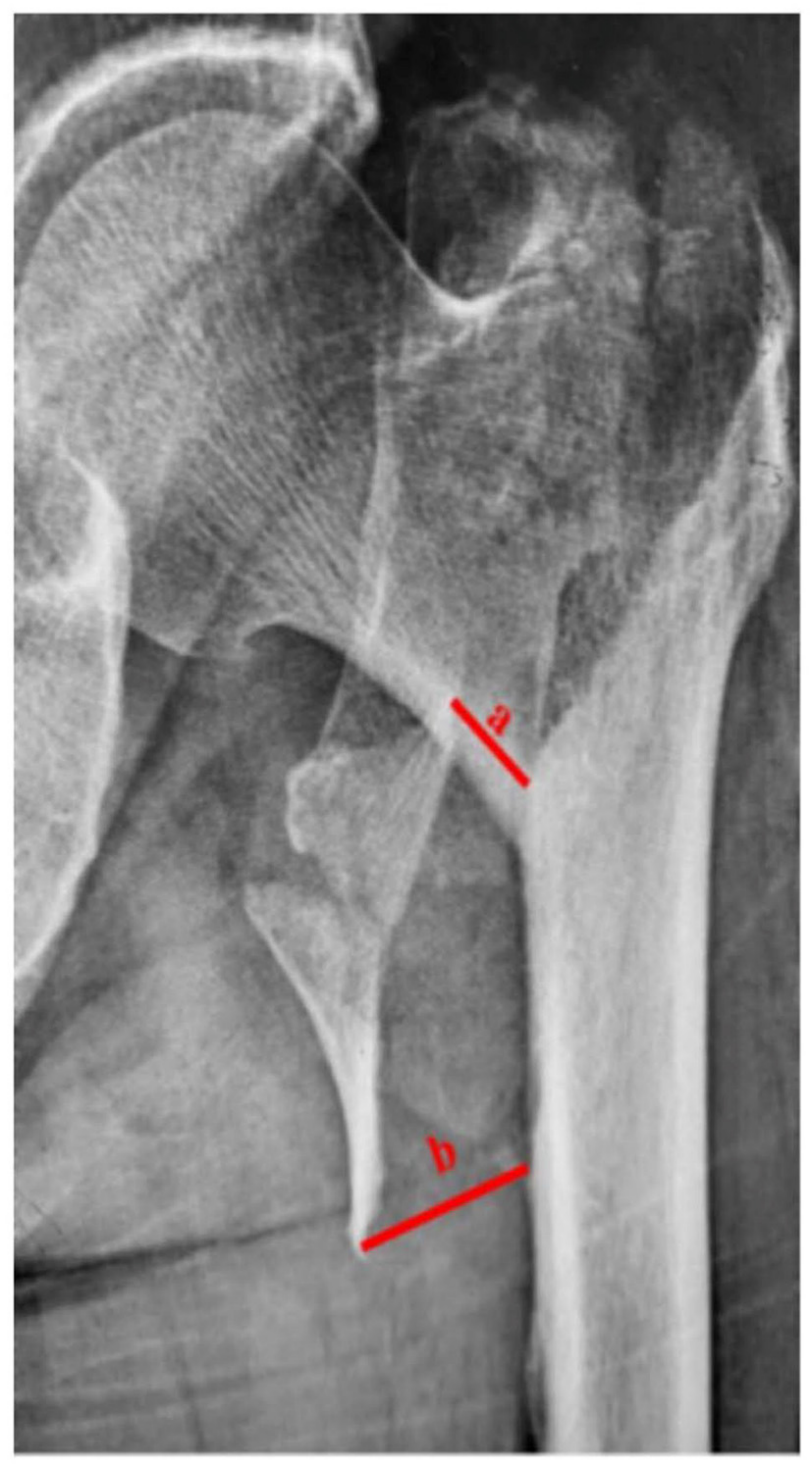
Distance a and b are defined as the lengths from the highest fracture site to the corresponding site at the lesser trochanter and the lowest fracture site to the corresponding site at the lesser trochanter, respectively. And the displacement distance of lesser trochanter is defined as the average of the distance a and distance b.

Based on anteroposterior X-ray films in all included studies, three studies set up the concrete cut-off of displacement distance of LT (DDLT, measurement method depicted in Figure 5), including 5 mm (12), 10 mm (22), and 20 mm (3) (Table 1). Besides, two other studies set up the concrete quantity and sizes of displaced LT. One was that three groups were built up based on the new classification for a medial wall in trochanteric fractures (17); the other study was that all patients were divided into five groups according to the quantity of LT fragment (<2, =2, and >2) and range of LT fragment in the femoral posteromedial wall (<75, ≥75%) (42).

As for the surgical protocols for trochanteric fractures with displaced LT in 880 patients, 196 (22.27%) patients' displaced LT were reduced and fixed while the rest (77.73%) were not. And 196 fixation devices consisted of 19 (9.69%) lag screws and 177 (90.31%) wires, of which 22 (12.43%) wires were bundled up as modified candy-package while bundling techniques of the rest of 155 (87.57%) wires were not mentioned. Besides, among 196 patients with displaced LT fixed, 58 (29.59%) cases underwent IM with wiring, 119 (60.71%) DHS with wiring, and 19 (9.69%) DHS with a lag screw.

### Surgical Outcomes of Hip Function

Among the included studies, eight studies reported postoperative hip function with Harris hip score (HHS) (43) or Western Ontario and McMaster Universities Osteoarthritis Index (WOMAC) (44), three reported femur NSA, two reported hip flexion strength, and one reported femoral neck shortening (FNS). It is obvious that HHS/WOMAC is the most concerned outcome indicator for orthopedic surgeons. Puram et al. (20) believed that HHS was such a representative because it reflected both function and symptoms of the hip, including pain, deformity, limp, shortening, and range of motion. According to HHS checklist (100 points), ≥90, excellent; 80–89, good; 70–79, fair; <70, poor (Table 2).

**Table 2:** Characteristics of outcomes of reducing and fixing lesser trochanter.

**References**	**HHS or WOMAC** **score**	**Complication** **incidence**	**Implant failure**	**Operative time, min**.	**Blood loss, ml**.	**Others**
Kim et al. (12)	45.4 (21–75) Pre-Trauma: 36.5 (19–59) (*p* = 0.087)	3/22	2	NR	NR	NR
Sun et al. (22)	A: 85.2 ± 7.9 B: 82.3 ± 7.6 C: 83.7 ± 8.9 (*p* = 0.374)	A: 2.4% B: 18.2% C: 5.6% (p=0.043)	A: 1 B: 3 C: 2	NR	NR	VAS^a^ score A: 0.4 (0–2) B: 1.1 (0–5) C: 0.5 (0–3) (*P* = 0.023)
Liu et al. (5)	A: 81.20 ± 3.32 B: 81.24 ± 3.35 (*p* = 0.622)	A: 8.33% B: 10.81% (*p* = 0.698)	NR	A: 46.2 ± 4.2 B: 50.4 ± 6.6 (*P* <0.05)	A: 107.03 ± 49.21 B: 133.96 ± 58.08 (*P* <0.05)	NR
Ye et al. (9)	91.80 ± 3.05	1/32	1	NR	NR	NR
Guo et al. (41)	A: acceptable: 83.3% B: acceptable: 58.1% (*p* = 0.049)	A:1/15 B:10/31	A: 2 B: 10	A: 58.4 ± 5.3 B: 186.3 ± 6.6 (*P* = 0.000)	A: 186.3 ± 6.6 B: 246.2 ± 8.7 (*P* = 0.000)	NR
Puram et al. (20)	A: 84.15 ± 8.65 B: 82.54 ± 6.60 (*p* = 0.392)	NR	NR	NR	NR	NR
Aprato et al. (19)	Modified A: 92.8 (SD 4.4) B: 96.8 (SD10.1) (*p* = 0.204)	NR	NR	NR	NR	Hip flexion strength: Neutral position (*p* = 0.034) 90° position (*p* = 0.008)
Schenkel et al. (3)	94.73 (36.73–100)	NR	NR	NR	NR	Hip flexion strength: 0° position (*p* = 0.498) 30° position (*p* = 0.587)
Li et al. (17)	NR	NR	A: (0.5%) B: (1.3%) C: (9.7%) (*p* = 0.001)	NR	NR	NR
Ren et al. (42)	NR	NR	NR	NR	NR	NSA^b^ change A: 5.16 ± 2.13° D: 6.92 ± 2.53° B+C+E: 10.13 ± 6.17° (*P* <0.025) FNS^b^ (mm) A: 4.97 ± 3.61 D: 5.41 ± 2.79 B+C+E: 12.27 ± 4.18 (*P* <0.01)

*HHS: Harris hip score, according to HHS checklist (100 points), ≥90, excellent; 80–89, good; 70–79, fair; <70, poor*.

*WOMAC: Western Ontario and McMaster Universities Osteoarthritis Index, a checklist of 24 questions. Lower scores indicate better joint function*.

a *VAS: visual analog scale (10 points), 0–2, excellent; 3–5, good; 6–8, fair; >8, poor*.

b *NSA, Neck-Shaft Angle. FNS, Femoral Neck Shortening*.

Out of eight studies that made a statistical analysis about HHS/WOMAC, five studies (5, 12, 19, 20, 22) demonstrated no statistical significance between/among groups, two studies (3, 9) verified that both fixation and non-fixation of displaced LT got “excellent” marks. One study (41) substantiated that fixation of displaced LT would get a higher mark (*p* = 0.049). It should be noticed that no statistical significance in HHS was observed between DHS with wiring (20) vs. IM nail with wiring (22) (*p* = 0.444) (Table 3).

**Table 3:** Statistics of the primary outcome indicator of included studies.

**Study name**	**Statistics for each study**			**Relative weight (%)**
	**Harris hip score (HHS)**	**Lower limit**	**Upper limit**	
**Non-fixed treatment group**				
Sun et al. (22)	83.9	82.1	85.7	34.56
Liu et al. (5)	81.2	80.3	82.1	22.23
Guo et al. (41)	73.3	66.8	79.8	15.67
Puram et al. (20)	84.7	81.5	87.9	12.90
Aprato et al. (19)	92.8	90.3	95.3	5.53
Schenkel et al. (3)	94.7	Not estimable	Not estimable	9.22
**Fixed treatment group**				
Sun et al. (22)	83.7	80.8	86.6	25.35
Guo et al. (41)	81.2	75.7	86.7	22.54
Puram et al. (20)	82.5	81.0	84.0	52.11

The NSA is a meaningful method to assess the collapse degree of the femoral head after fracture, which is 127 degree (range, 110–140 degrees) in adults (45). Besides, recently orthopedists prefer to use the term “telescoping” to quantify the degree of FNS after fracture, of which the value is negatively correlated with the stability of the hip joint (42). Thus, orthopedic surgeons try to reduce and fix the fracture fragments to their original anatomical position in case of substantial changes of NSA and FNS leading to instability of the femur-implant structure, even implant failure. However, the surgical protocol of DHS with wiring (20, 41) had less but not significant influence on postoperative NSA changes (*p* > 0.05) compared to that of non-fixation for LT. Nevertheless, one study (42) demonstrated that larger (≥75% area of the posteromedial wall) and much more comminuted (≥2) LT fragments could result in statistically significant changes of NSA (*p* < 0.025) and FNS (*p* < 0.01).

As for the hip flexion strength, Aprato et al. (46) believed that displaced LT implication could significantly reduce hip flexion strength compared to that of the trochanteric fractures without displaced LT in 90 degree position, neutral position, and Figure 4 position (*p* = 0.008, *p* = 0.034, *p* = 0.034, respectively). Although Schenkel et al. (3) demonstrated that on comparison with the uninjured hip side, non-fixation for LT had no significant influence on hip flexion strength in 0 and 30 degrees position (both *p* > 0.05).

### Complications

Various complications associated with displaced LT were reported in 10 studies including implant failure, severe thigh pain, loss of reduction, and hip varus deformity. However, only six studies reported complications and out of which two studies (17, 22) demonstrated that much bigger displaced LT (type 3 or its displacement distance ≥1 cm) fragments had significant differences in complication incidence to that of the inferiors (*p* = 0.001, *p* = 0.043, respectively). Besides, implant failure is seriously necessary for the revision operation. Only one study (17) reported that this severe complication was defined: (1) subsequent fracture, (2) fracture nonunion, (3) cut-out, (4) implant or screw breakage, (5) progressive fracture displacement, and (6) lateral protrusion of the blade or screw. In the meanwhile, they also demonstrated that LT of type 3 (one type in the newly reported classification of the medial wall in trochanteric fractures mentioned above) had a significant incidence of implant failure to that of other types (*p* = 0.001). What's more, it was investigated that large (≥1 cm) displaced LT without fixation could result in significantly unbearable thigh pain (*p* = 0.023) (22) (Table 4).

**Table 4:** Statistics of the secondary outcome indicator of included studies.

**Study name**	**Statistics for each study**			**Relative weight (%)**
	**Complication incidence (%)**	**Lower limit**	**Upper limit**	
**Non-fixed treatment group**				
Sun et al. (22)	20.60	10.90	29.10	15.59
Liu et al. (5)	9.30	0.50	16.20	9.98
Guo et al. (41)	32.26	16.60	48.10	7.07
Li et al. (17)	2.50	0.80	4.20	67.36
**Fixed treatment group**				
Qi et al. (22)	5.60	−1.90	13.00	29.51
Guo et al. (41)	6.67	−2.10	14.60	26.23
Kim et al. (12)	13.64	−0.70	28.00	18.03
Ye et al. (9)	3.13	−2.90	9.20	26.23

### Cost of Reduction and Fixation for Displaced LT

Only two studies (5, 41) reported the differences in blood loss and operative time due to different fracture types and surgical protocols between subgroups. It was reported that both using PFNA treatments, the operative time, and blood loss were significantly elevated in trochanteric fractures with displaced LT than that of those with intact LT (both, *p* < 0.05) (5). Moreover, between LT fixed group and not fixed group the former was demonstrated that would significantly augment the two costs (both, *p* < 0.05) (41).

## Discussion

To the best of our knowledge, a review of LT is rare and the current systematic review on this subject is the first. In the current study, the primary finding was that during 2013–2020's orthopedic surgeons preferred not to deal with the displaced LT (636 cases of LT non-fixation in 880 cases involving displaced LT, 72.7%). But if they did deal, they preferred to choose the protocols of DHS with wires (60.71%) than IM nail with wires (29.59%), and DHS with lag screw (9.69%).

Moreover, we analyzed some significant elements and surgical protocols that future orthopedic surgeons should notice in operations for trochanteric fractures with kinds of displaced LT. In 10 included studies, HHS was the most used and reported clinical instrument to assess patients' postoperative hip function in multiple aspects, as the primary outcome indicator in the current study as well. After evaluating the correlation between HHS and surgical protocols, it was not surprised that whether to fix displaced LT or not, no statistical significance in HHS was observed, and both resulted in excellent/good HHS marks (*p* > 0.05), which was consistent with the conventional operation selection trend that most orthopedists preferred not to reduce or fix LT fragment. However, one study reported statistical significance in HHS between subgroups (*p* = 0.049) (41), and we analyzed this might result from restrictive fracture types of distribution and bias. Moreover, we observed that protocols of DHS/IM nail and wires/lag screw all got excellent/good HHS marks and no significant differences among these methods were found. In the meanwhile, we observed that when the quantity of comminuted LT fragments was ≥2 and the range of LT fragments in the posteromedial wall was ≥75%, the differences of complications associated indicators, femoral NSA change, and FNS, reached statistical significance (*p* < 0.01, *p* < 0.025, respectively). Besides, one biomechanical study (8) demonstrated that specific protocol, such as PFNA with LT fixation, could significantly reduce FNS. Therefore, we analyzed that severely comminuted LT might exert a negative role in stability of the femur-implant structure, and protocol of PFNA with wiring LT would have more benefits. But specific surgical protocol should be further individually ascertained after assessing bony quality and fracture types of various patients. After exclusion of possible influence due to uneven distribution of fracture types and complex hip strength compensatory mechanism, we analyzed hip flexion strength, a complication associated indicator as well, was not significantly affected by displaced LT nor the various surgical protocols, which was consistent with HHS (Table 5).

**Table 5:** Statistical significance of two outcome indicators of included studies.

**Study name**	**Statistics significance**					**Relative weight (%)**
	**Statistical difference value**	**Lower limit**	**Upper limit**	**Standard error**	**Significance level**	
**The primary outcome indicator: HHS**						
Sun et al. (22)	−0.200	−3.483	3.083	1.656	0.904	40.07
Guo et al. (41)	7.900	−0.826	16.626	4.368	0.075	23.10
Puram et al. (20)	−2.200	−5.381	0.981	1.604	0.173	36.82
**The secondary outcome indicator: complication incidence**						
Qi et al. (22)	14.444%	2.7%	26.2%	0.060	0.048	63.43
Guo et al. (41)	26.103%	8.3%	43.9%	0.091	0.008	36.57
**Subtotal:**	13.188%	5.6%	20.8%	0.039	0.002	100

Complication was the other vital parameter to weigh the feasibility of carrying out a reduction and fixation procedure for displaced LT during operation, as the secondary outcome indicator in the current study as well. We analyzed that the incidence of complication was significantly elevated when the displaced LT featured as DDLT ≥2 cm or type 3, especially the implant failure in the latter situation.

Previous studies had demonstrated that in unstable trochanteric fractures reduction and fixation for small and large LT fragments could increase the mechanical stability by 17 and 57%, respectively (6). It also had been substantiated that large LT, as well as its extent calcar, exerted a vital influence on redistributing stress in the proximal femur by increasing load in the anterolateral wall and decreasing the load in the posteromedial wall (4). Besides, fixation for displaced LT could exert a buttress effect on the posteromedial cortex to get initial stability, which could allow patients early ambulation (47). It is widely identified by clinical physicians that large posteromedial bone fragments with large value of displacement distance need to be surgically fixed, but no consensus is reached for the concrete degree of this “large”(4, 17, 22). We observed that four included studies had implied how large of displaced LT should be noticed: DDLT ≥ 10 mm (3), ≥ 20 mm (22), a quantity of comminuted LT fragments ≥2 with a range of displaced LT in posteromedial wall ≥75% (42), and type 3 (17). Moreover, Zhang et al. (4) divided the LT fragments into three types: dislocated but intact LT, LT maintaining a continuation with greater trochanter and quantity of comminuted LT fragments >3. From which, they recommended reduction and fixation for the former two types, but the last type was not for difficulties in present techniques or increased intraoperative risks. Owing to the inadequate reports of primary and secondary outcome indicators in included studies, overall statistical analysis was hard to perform. Therefore, we proposed a novel classification about cut-offs of DDLT and the sizes of displaced LT to assess when prefer to get displaced LT fixed: (a1) DDLT <1 cm, preferring not to reduce or fix displaced LT, (a2) 1 cm ≤ DDLT < 2 cm, demanding further individual judgment, (a3) DDLT ≥ 2 cm, preferring to reduce and fix displaced LT; (b1) quantity of comminuted LT fragments <2 or range of LT fragments in medial wall <75%, preferring not to reduce or fix displaced LT, (b2) quantity of comminuted LT fragments ≥2 and range of LT fragments in medial wall ≥75%, preferring to reduce and fix displaced LT; (c1) fracture line of LT fragments not reaching the midline of the posterior wall, preferring not to reduce or fix displaced LT, (c2) fracture line of LT fragments reaching or exceeding the midline of the posterior wall, preferring to reduce and fix displaced LT.

### Limitations

Systematic assessment of included studies revealed several limitations that more than half of them reported inadequate main outcome indicators, thus meta-analysis and overall statistical analysis were limited to perform. Moreover, the topic “postoperative outcome” of patients with trochanteric fractures generally was not affected by displaced LT alone. More elements, like fracture line involving femoral lateral wall, osteoporosis, anatomical reduction, and surgical devices selection, etc., were previously demonstrated that played pivotal roles in postoperative outcomes (4), which means that these confounding parameters with inadequately reporting might delay proper evaluation of efficacy frequency and severity of adverse events. Therefore, these limitations should be further systematically investigated in the future studies.

## Conclusion

In the current study, we suggested that it was beneficial to reduce and fix the displaced LT when one of conditions below occurred: DDLT ≥2 cm; quantity of comminuted LT fragments ≥2 and range of LT fragments in medial wall ≥75%; and fracture line of LT fragments reaching or exceeding the midline of the posterior wall. Besides, we didn't find any significant difference in postoperative outcomes between the two protocols PFNA with LT wires and DHS with LT wires.

## Data Availability Statement

The original contributions presented in the study are included in the article/supplementary material, further inquiries can be directed to the corresponding author.

## Author Contributions

Y-HD and A-LY had the idea for the article. A-LY and WM performed the literature search, data analysis, and the first draft. Y-HD, J-GW, Y-QH, H-FN, and H-LL critically revised and commented on previous versions of the manuscript. All authors read and approved the final manuscript.

## Funding

This study was funded by the Highlevel Talent Special Fund of the Minhang District of Shanghai (File No. 5-1), which is received for open access publication fees.

## Conflict of Interest

The authors declare that the research was conducted in the absence of any commercial or financial relationships that could be construed as a potential conflict of interest.

## Publisher's Note

All claims expressed in this article are solely those of the authors and do not necessarily represent those of their affiliated organizations, or those of the publisher, the editors and the reviewers. Any product that may be evaluated in this article, or claim that may be made by its manufacturer, is not guaranteed or endorsed by the publisher.

## Abbreviations

DDLT: displacement distance of LT
NSA: neck-shaft angle
FNS: femoral neck shortening
LTFQ: quantity of comminuted lesser trochanter fragments
LTFR: range of lesser trochanter fragment in medial wall
VAS: visual analog scale.
